# Validity of Nutritional Screening Tools for Hospitalized Children

**DOI:** 10.1155/2014/143649

**Published:** 2014-09-14

**Authors:** Nathania Wonoputri, Julistio T. B. Djais, Ina Rosalina

**Affiliations:** Department of Child Health, Universitas Padjadjaran, Dr. Hasan Sadikin General Hospital, Jalan Pasteur No. 38, Bandung 40161, Indonesia

## Abstract

*Background*. Malnutrition in hospitalized children can be prevented if children with risk of malnutrition are identified. Every hospital is recommended to have a standard nutritional screening tool. Numerous simple screening tools have been developed, namely Paediatric Yorkhill Malnutrition Score (PYMS), Screening Tool for the Assessment of Malnutrition in Paediatrics (STAMP), and Screening Tool for Risk on Nutritional Status and Growth (STRONG-kids). None has been accepted as a universal tool. Our study aims to determine the best screening tools compared to Subjective Global Nutrition Assessment (SGNA), an assessment tool which is more complex as our gold standard. *Methods*. This diagnostic study involved 116 patients aged 1–15 years. Three screening tools and SGNA were examined to each subject. Statistical analysis was used to determine sensitivity, specificity, and likelihood ratio (LR) by results from screening tools divided into low and moderate-high risk of malnutrition compared to results from SGNA divided into no and moderate-severe malnutrition. *Results*. PYMS showed superior agreement to SGNA resulting in sensitivity 95.32%, specificity 76.92%, positive LR 4.13, and negative LR 0.061. STAMP resulted in sensitivity, specificity, positive LR, and negative LR, respectively, as 100%, 11.54%, 1.13, and 0 and STRONG-kids resulted in 100%, 7.7%, 1.083, and 0. *Conclusion*. PYMS was the most reliable screening tool.

## 1. Introduction

Substrates such as food and vitamins are required by children as the materials in daily metabolism. Increased cost of growth leads to risks of malnutrition especially in hospitalization [1]. Malnutrition has been associated with increased mortality, morbidity, length of stay, and, eventually, cost itself [1–6]. Previous studies reported prevalence of malnutrition in pediatric hospitals ranging from 15 to 50% [3, 5–9]. The European Society for Clinical Nutrition and Metabolism (ESPEN) [10], American Society for Parenteral and Enteral Nutrition (ASPEN) [1, 8], and European Society for Paediatric Gastroenterology, Hepatology and Nutrition (ESPGHAN) [6] recommend nutritional screening, which is rapid and simple, to be used to determine patients who are nutritionally at risk. Nutritional assessment using an assessment tool is then indicated for children nutritionally at risk, facilitating early detection of subsequent nutrition deterioration and finally adequate therapy. An assessment tool is a complete clinical assessment of nutritional status including several steps with detailed medical and dietary history and physical examination including anthropometric, body composition measurement, and laboratory data. The process is lengthy and more time-consuming making it impossible to evaluate every child admitted to the hospital [1, 11]. An assessment tool that has been adapted for children, Subjective Global Nutritional Assessment in Children (SGNA), has shown its ability to identify malnourished children, identifying those at higher risk of nutrition-associated complications, and prolonged hospitalizations [12, 13]. The usage of SGNA has also been validated in our hospital [14]. However, assessment tools are more complex and time-consuming [15]. Therefore, a nutritional screening tool is needed to evaluate every child admitted to the hospital [10].

Numerous screening tools have been developed in previous years, but no universally accepted screening tools are available [9–11]. Paediatric Yorkhill Malnutrition Score (PYMS) was developed and validated in United Kingdom for hospitalized children aged 1–16 years old [16]. Screening Tool for the Assessment of Malnutrition in Paediatrics (STAMP) was also developed and validated in the United Kingdom for hospitalized children [17]. Screening Tool for Risk on Nutritional Status and Growth (STRONG-kids) was developed and validated in Dutch hospitals screening children between 1 month and 16 years [18]. Validation of these three screening tools has never been conducted in our hospital. Therefore, our study aims to determine concurrent validity between the three nutritional screening tools to SGNA, an nutritional assessment tool in Dr. Hasan Sadikin General Hospital, Bandung, Indonesia, a tertiary general hospital.

## 2. Materials and Methods

This diagnostic study was conducted from January to February 2014 in Department of Child Health, Dr. Hasan Sadikin General Hospital, Bandung, Indonesia. This study has been approved by the Health Research Ethics Committee, Faculty of Medicine, Padjadjaran University, Bandung, Indonesia. The inclusion criteria were patients between 1 and 17 years old, admitted to the pediatric ward for at least 24 hours and whose parents had agreed to participate in the study.

Sampling technique used was consecutive sampling. Patient's characteristic data were recorded for gender, age, underlying disease, weight, height, and body mass index. Anthropometry measurements were taken in a standardized way and then plotted on the WHO Growth Chart or WHO Growth Reference to determine malnutrition. Our study used the definition of acute and chronic malnutrition according to WHO [19]. Acute malnutrition was defined as a weight for height (WFH) SD score lower than −2. Body mass index for age was used if information was not available. Chronic malnutrition was defined as height for age (HFA) SD score lower than −2.

Three screening tools (STAMP, PYMS, and STRONG-kids) and one assessment tool (SGNA) were evaluated in each participant by a single operator. The STAMP [20] and PYMS [2] tools consist of 5 steps with questions and points for every answer yielding a sum total which is divided into 3 categories: high, medium, and low risk of malnutrition. The STRONG-kids tool consists of 4 questions with an allocated score resulting in the same 3 categories [18]. The SGNA assessment tool consisted of nutrition-focused medical history and physical examination yielding an overall ranking as normal, moderate, and severe malnutrition [21]. Statistical analyses were used to determine sensitivity, specificity, and likelihood ratio by defining 2 × 2 tables, which compares the results from low and moderate-high risk of malnutrition categories to the results from no malnutrition and moderate-severe malnutrition categories. Kappa value was used to determine the ability of each of the screening tools to pick up acute and chronic malnutrition according to WHO. Results from low and medium-high risk are compared to normal and acute/chronic malnutrition for determining kappa value.

## 3. Results

Subject characteristics of 116 participants are shown in Table 1. A total 116 patients participated in the study ranging from 1 to 15 years old. The malnutrition prevalence in this study is found to be 28.44%. A total of 33 children which are considered to be malnourished based on WHO criteria were grouped based on their underlying disease as shown in Table 2, most of who suffered from oncology disorder.

Table 3 showed the kappa value used to determine the ability of each screening tool and SGNA to pick up acute/chronic malnutrition. PYMS was the screening tool that showed best agreement with the anthropometric measurement of acute and chronic malnutrition.

The sensitivity, specificity, and likelihood ratio between nutritional screening tools and SGNA are shown at Table 4. PYMS showed superior results than other nutritional screening tools evaluated.

## 4. Discussion

As shown in Table 1, malnutrition prevalence found for our study is 28.44%. This value is still in the range from previous reports, which ranges from 15 to 50% [3, 5–9, 22]. Most of the children that are found to be malnourished are the children with oncology disorder, followed by infectious disease, and neurological disorder with infectious disease (Table 2). Malnutrition in patients with oncology disorder is common [23], while children with infectious disease were found to be one of the largest proportions of malnourished patients as in previous study [22]. Poor growth and nutritional status are often seen in children with neurological disorder [23], especially with combined diagnosis [22].

Anthropometric measurements are used for assessing nutritional status worldwide [9]. By applying the definition of acute and chronic malnutrition from WHO based on anthropometric measurements, our study tried to determine the ability of each nutritional screening tool to pick up the types of malnutrition. In Table 3, PYMS showed superior results compared with other nutritional screening tools based on its kappa value.

Similar result is also shown by sensitivity, specificity, and LR parameters. When being compared with SGNA as the gold standard, PYMS is the best screening tool that is used in our hospital. The PYMS showed sensitivity 95.31% implying false negative 4.69% and specificity 76.92% implying false positive 23.08%. The STAMP showed sensitivity 100% implying no false negative, however specificity 11.54% implying false positive 88.46%. The STRONG-kids showed sensitivity 100% implying no false negative, however specificity 7.7% implying false positive 92.3%. PYMS showed the smallest false positive. Estimation of the probability of the disease in individual patients is described by the predictive values [24]. The PYMS showed both high positive (83.56%) and negative predictive values (93.02%). Even though STAMP and STRONG-kids showed very high negative predictive value (100%), they showed positive predictive value with lower value than that of PYMS. Sensitivity, specificity, positive predictive value, and negative predictive value vary according to prevalence; hence a stable characteristic of a diagnostic test is needed, namely, likelihood ratio that gives strength of the test [25]. Likelihood ratio of PYMS concluded fair strength from the positive LR (4.13) and excellent strength from negative LR (0.061). Positive LR of STAMP concluded useless strength (1.13) and excellent strength from negative LR (0). Positive LR of STRONG-kids concluded useless strength (1.083) and excellent strength from negative LR (0). Eventually, usage of STAMP and STRONG-kids might lead to overdiagnosis; therefore, PYMS was the best screening tool without too much overdiagnosis according to our study.

Until now, there is no accepted gold standard for the assessment of the nutritional status of a child [9]. Based on our knowledge, no previous study has used SGNA as a gold standard to compare screening tools in children. There is a lot of debate among professionals on how to validate screening tools, especially in validating a nutritional screening tool if it can predict current nutritional status. Many have validated their screening tools with full dietetic assessment. However, it is questionable if this is the gold standard, especially as not all countries have dieticians and their role may vary depending on the country [9], as in Indonesia. In this study, SGNA is chosen as the gold standard. SGNA was initially designed as a screening tool. The SGNA consists of both subjective and objective components in a detailed questionnaire and complete physical examination; then each child is classified as well-nourished, moderately malnourished, or severely malnourished. Completion of SGNA is lengthy and time-consuming [11]. More in-depth than nutrition screening tool, SGNA is now used to assess nutritional status of children who may be at risk of malnutrition such as those who are hospitalized [21]. SGNA has been evaluated in 175 children admitted for a major surgical procedure. Malnourished children had higher rates of nutrition-associated complications such as infectious complications and postoperative length than well-nourished children [12]. Our hospital has also evaluated SGNA in 2010. SGNA was evaluated in 320 children at our hospital showing that malnourished children had longer length of hospital stay than well-nourished children [14]. Since then, SGNA has been the assessment tool in our hospital. Therefore, our study chose SGNA as our gold standard.

All previous studies showed comparisons of nutritional screening tools with full nutritional assessment as their gold standard. Gerasimidis et al. [2] compared STAMP, PYMS, and SGNA to full nutritional assessment. The study included 2,174 children from pediatric and surgery ward concluding that PYMS with sensitivity 85% and specificity 87% is an acceptable screening tool for identifying children at risk of malnutrition without producing unmanageable numbers of false-positive cases. Children from cardiology, renal, orthopaedic specialties, and critical care were not included. A 2 × 2 table was made from the results of STAMP, PYMS, and SGNA to determine sensitivity and specificity, which divided the result into low-medium risk and high risk, compared to full nutritional assessment [2]. Our study included all patients in the pediatric ward. However, no patients were listed for surgery in our study. Our study divided the 2 × 2 table differently into low and medium-high risk to determine sensitivity and specificity. Another study conducted by Moeeni et al. [13] comparing PYMS, STAMP, and STRONG-kids to full nutritional assessment which included 119 children in Iran suggested that STRONG-kids was the most useful and reliable tool. Weight for height* z*-score correlated with all three tools (*P* < 0.001), but only STRONG-kids correlated with height for age* z-*score (*P* = 0.04). The STRONG-kids detected more children with moderate undernutrition compared to PYMS (*P* = 0.0001) and STAMP (*P* = 0.05). High risk determined by STRONG-kids was also associated with length of stay (*P* = 0.004). PYMS was superior in detection of the severely undernourished children compared to STRONG-kids (*P* = 0.003) [13]. Another study conducted by Moeeni et al. [26] that evaluated 162 children with PYMS, STAMP, and STRONG-kids to full nutritional assessment in New Zealand recommended that STRONG-kids was the most reliable tool. Only high and moderate risk children using PYMS (*P* = 0.01 and *P* = 0.001, resp.) and STRONG-kids (*P* = 0.003 and *P* = 0.002, resp.) had significantly longer admissions than low risk group [26]. Both studies conducted by Moeeni et al. [13, 26] were cross-sectional studies comparing hospitalized and nonhospitalized children. The study recorded the length of stay and excluded oncology disorder in their population. However, in this diagnostic study the oncology patients were included.

According to ESPEN, screening tools are used to detect protein and energy undernutrition and/or to predict whether undernutrition is likely to develop/worsen under the present and future conditions of the patient/client. Therefore, screening tools should consist of four main principles.

1. The current condition can be described in the height and weight allows the calculation of body mass index.
2. Is the condition stable? This concerns any recent weight loss that can be obtained from patient's history or previous measurements in medical records.
3. Will the condition worsen? This question might be answered by asking whether food intake has been decreased up to the time of screening and if so by approximately how much and how long.
4. Will the disease process accelerate nutritional deterioration? This item concerns the underlying disease process which may increase nutritional requirement due to the stress metabolism associated with the severity of the underlying disease, causing nutritional status to worsen more rapidly or to develop a poor nutritional status.

Variables 1–3 should be included in all screening tools, while variable 4 is relevant mainly in hospital settings. Each variable must be given a score in every screening tool, thereby quantifying the degree of risk and a direct course of action [10]. Each nutritional screening tool used in our study is compared according to the main principles based on ESPEN of a screening tool and their aims showed in Table 5. STRONG-kids also used subjective clinical assessment and is the only screening tool that does not include height and weight which some may consider it as its disadvantages [3, 27]. Pediatricians may believe that they can recognise a malnourished child but the fact does not always agree with this. Reproducibility in clinical assessment of nutritional status is poor in a study conducted by Cross et al. [28], especially in assessing the more severely malnourished children. Clinical evaluation of nutritional status alone is inadequate for accurate assessment and anthropometry is prominent [27]. The screening tool PYMS does not include the impact of underlying disease. The other two screening tools included lists of underlying disease in their components [13], which has been considered as a disadvantage [11, 13]. However, it must be noted that PYMS still asked about condition effect on nutrition whether due to decreased intake caused by orofacial trauma or severe nausea, increased gut loss, increased energy requirements, or intention to perform major abdominal surgery causing minimal intake [16]. This was emphasized by a review that concluded that PYMS also evaluates disease severity [9].

In conclusion, we recommend PYMS as the most reliable screening tool in Dr. Hasan Sadikin General Hospital in Bandung.

## Figures and Tables

**Table 1:** Subjects characteristics of the participants.

Characteristics	Total (*n* = 116)	Percentage (%)
Age		
1–≤5 years	42	36.21
5–≤10 years	45	38.79
>10 years	29	25
Gender		
Male	66	56.90
Female	50	43.10
Underlying disease		
Oncology disorder	50	43.10
Renal disease	12	10.34
Infection disease	17	14.65
Bleeding diathesis	6	5.17
Neurological disorder	3	2.59
Neurological disorder with infection disease	6	5.17
Neurological disorder with respiratory condition	3	2.59
Cardiac disease	3	2.59
Immunological disorder	2	1.72
Endocrinology disease	1	0.86
Gastrointestinal disease	5	4.31
Respiratory disorder	8	6.90
Acute malnutrition (WFH < −2 SD)		
Malnutrition prevalence	33	28.44
Moderate malnutrition	17	14.66
Severe malnutrition	16	13.79
Chronic malnutrition (HFA < −2 SD)		
Stunted	24	20.69
Severely stunted	24	20.69

**Table 2:** Malnutrition prevalence based on the underlying disease.

Underlying disease	Severe malnutrition	Moderate malnutrition	Total (*n* = 33)
Oncology disorder	6	5	11
Infection disease	3	6	9
Neurological disorder	1	1	2
Neurological disorder with infection disease	2	1	3
Neurological disorder with respiratory condition	2	0	2
Gastrointestinal disease	1	1	2
Renal disease	1	0	1
Cardiology disease	0	1	1
Respiratory disorder	0	2	2

**Table 3:** Kappa value between each of the screening tools and SGNA compared to acute/chronic malnutrition.

Tools	Kappa value (95% CI) for acute malnutrition (wasting)	Kappa value (95% CI) for chronic malnutrition (stunted)
PYMS	0.348 (0.191–0.506)	0.125 (0–0.299)
STAMP	0.018 (0–0.140)	0 (0–0.140)
STRONG-kids	0.028 (0–0.149)	0 (0–0.144)

**Table 4:** Sensitivity, specificity, and likelihood ratio between nutritional screening tools and SGNA.

	Sensitivity (95% CI)	Specificity (95% CI)	Positive predictive value (95% CI)	Negative predictive value (95% CI)	Positive LR (95% CI)	Negative LR (95% CI)
PYMS	95.31 (0.87–0.98)	76.92 (0.63–0.86)	83.56 (0.73–0.9)	93.02 (0.81–0.97)	4.13 (2.507–6.804)	0.061 (0.02–0.186)
STAMP	100 (0.94–1)	11.54 (0.05–0.23)	58.2 (0.48–0.67)	100 (0.61–1)	1.13 (~)	0 (0–~)
STRONG-kids	100 (0.94–1)	7.7 (0.03–0.18)	57.14 (0.479–0.659)	100 (0.51–1)	1.083 (1.036–1.222)	0 (0–0.757)

LR: likelihood ratio.

**Table 5:** Comparison of screening tools based on 4 main principles of a screening tool according to ESPEN [9, 10].

Tools	Main principles according to ESPEN				Aim of each screening tool		
	Current nutritional status	Weight loss	Reduced intake	Disease severity	Identify nutritional status	Identify need for nutritional intervention	Predict clinical outcome without intervention
PYMS	*√*	*√*	*√*	√*	*√*	*√*	*√*
STAMP	*√*		*√*	*√*	*√*	*√*	
STRONG-kids	*√* ^ #^	*√*	*√*	*√*		*√*	*√*

^#^Current nutritional status by STRONG-kids is not evaluated by BMI but by subjective clinical assessments.

∗Disease severity in PYMS is not evaluated based on underlying disease that has nutritional implications as in STAMP or STRONG-kids, but on whether the child's nutrition will be affected by recent condition for at least the next week.
